# Genetic and transcriptional profiles of ammonia oxidizing communities in Bohai sediments: abundance, activity, and environmental correlations

**DOI:** 10.3389/fmicb.2025.1611213

**Published:** 2025-05-27

**Authors:** Yining Jiang, Xue Lou, Mingyang Wang, Minggang Zheng, Zhiyao Wang, Hui Chen

**Affiliations:** ^1^College of Environment and Safety Engineering, Qingdao University of Science and Technology, Qingdao, China; ^2^Research Center for Marine Ecology, First Institute of Oceanography, State Oceanic Administration, Qingdao, China; ^3^Australian Centre for Water and Environmental Biotechnology, The University of Queensland, Brisbane, QLD, Australia

**Keywords:** ammonia oxidizing archaea, ammonia oxidizing bacteria, complete ammonia oxidizers, ecological distribution, influencing factors

## Abstract

Ammonia oxidation, a crucial part in nitrogen cycle, is thought to be jointly driven by ammonia-oxidizing archaea (AOA), ammonia-oxidizing bacteria (AOB), and complete ammonia oxidation (comammox) in the ocean. However, the spatial distribution of these three ammonia-oxidizing microorganisms in the marine sediments, especially at the transcriptional level, remains underexplored. This study utilizes quantitative PCR and activity experiments to quantify the *amoA* gene of three ammonia oxidizers at both DNA and RNA levels, measure their potential nitrification rate, and assess their relative contribution to ammonia oxidation in the marine sediments in Bohai region in China. Further, we analyzed their correlations with key environmental factors. In the marine sediments of Bohai, the transcript abundance of AOA, AOB, and comammox *amoA* genes ranged from 7.31 × 10^2^ to 9.82 × 10^4^, 5.77 × 10^3^ to 3.98 × 10^4^ and 1.07 × 10^4^ to 5.44 × 10^4^ copies g^−1^ dry sediment, respectively. The results revealed that TN and TOC had significant effects on total *amoA* gene abundance and transcript abundance for all ammonia oxidizers. Besides, the relative contribution of AOB to ammonia oxidation was greater than that of AOA and comammox based on activity measurement, likely due to nitrate nitrogen and total nitrogen. Our study demonstrated that RNA-based *amoA* abundance and activity measurements can accurately reflect the spatial variations of ammonia oxidizers in Bohai sediments.

## 1 Introduction

The ocean, which sustains human survival and development and covers approximately 71% of the Earth’s surface, plays a crucial role in maintaining the global ecosystem. Ammonia oxidation is one of the key processes in the marine nitrogen cycle. Traditionally, it is divided into two consecutive steps (Winogradsky, 1890). The first step involves the conversion of NH_4_^+^ (the lowest valence state of the element nitrogen) to NO_2_^−^, which is also the rate-limiting stage of nitrification. This process is carried out by ammonia-oxidizing archaea (AOA) (Könneke et al., 2005) and ammonia-oxidizing bacteria (AOB) (Purkhold et al., 2000). The second step is the oxidation of NO_2_^−^ to NO_3_^−^ (the highest valence state of the element nitrogen) by nitrite-oxidizing bacteria (NOB) (Winogradsky et al., 1890). However, the discovery of comammox by Daims et al. (2015) and Van Kessel et al. (2015) confirmed the existence of complete ammonia oxidizers, predicted by Costa et al. (2006), which perform the one-step process from NH_4_^+^ to NO_3_^−^, challenging the traditional ammonia oxidation process.

Understanding the driving factors of ammonia oxidation in the environment is of great importance, as it contributes to understanding of nitrogen cycle and helps reduce the release of the greenhouse gas nitrous oxide (N_2_O) and nitrate (NO_3_^−^). The ammonia monooxygenase gene servers as a valuable indicator for the rate-limiting step nitrification and a possible indicator of potential N mineralization (Nelson et al., 2015). Therefore, the abundance of microbial functional gene and activity of ammonia oxidizers can be used to reveal the characteristics of the nitrogen cycle in marine ecosystem. Yet, fundamental questions remain regarding the activity, abundance of DNA and RNA, as well as contribution to ammonia oxidation in relations to environmental factors.

To date, the widespread presence of ammonia-oxidizing microorganisms has been confirmed in soils (Sun et al., 2022), intertidal zones (Zhao et al., 2024), estuarine sediments (Beman and Francis, 2006) and engineered ecosystems (Lu et al., 2016). However, research in marine ecosystems is still insufficient, with most studies not investigating the impact and role of comammox in the ocean. Furthermore, the focus has been on the quantitative analysis of the total abundance of ammonia oxidizers at the DNA level, which includes inactive cells (dead or dormant) (Allison et al., 2010). This approach does not accurately reflect the relationship between the function and abundance of ammonia oxidizers. Although RNA-based analyses have its shortcomings, such as its detection limitation and manifold factors that may affect nitrification rates (Li et al., 2022), it could better reflect the relationship between abundance and activity.

Previous studies have shown that ammonia oxidizers and environmental factors in soils (Rodriguez et al., 2021), bay (Sun et al., 2024), freshwater (Tang et al., 2025), artificial ecosystems (Fujitani et al., 2020) are closely related. For example, ammonia has consistently been a driving factor for the abundance and diversity of oxidizers, including comammox (Shi et al., 2018). In the soil ecosystem, comammox prefers to inhabit environments with low ammonia-nitrogen level and outcompetes other ammonia oxidizers. To date, the correlation between environmental factors and the abundance and activity of ammonia-oxidizing microorganisms in marine ecosystems remain to be studied. The Bohai Sea, with an average water depth of 18 m (Zeng et al., 2015), receives abundant nutrients from the Yellow River, and is closely linked to human activities. Exploring the abundance, activity, and environmental correlations of ammonia-oxidizing microorganisms in this area is crucial for understanding N cycling in marine ecosystem and mitigating anthropogenic environmental impacts.

## 2 Materials and methods

### 2.1 Site description and collection of sediment samples

**Figure 1:**
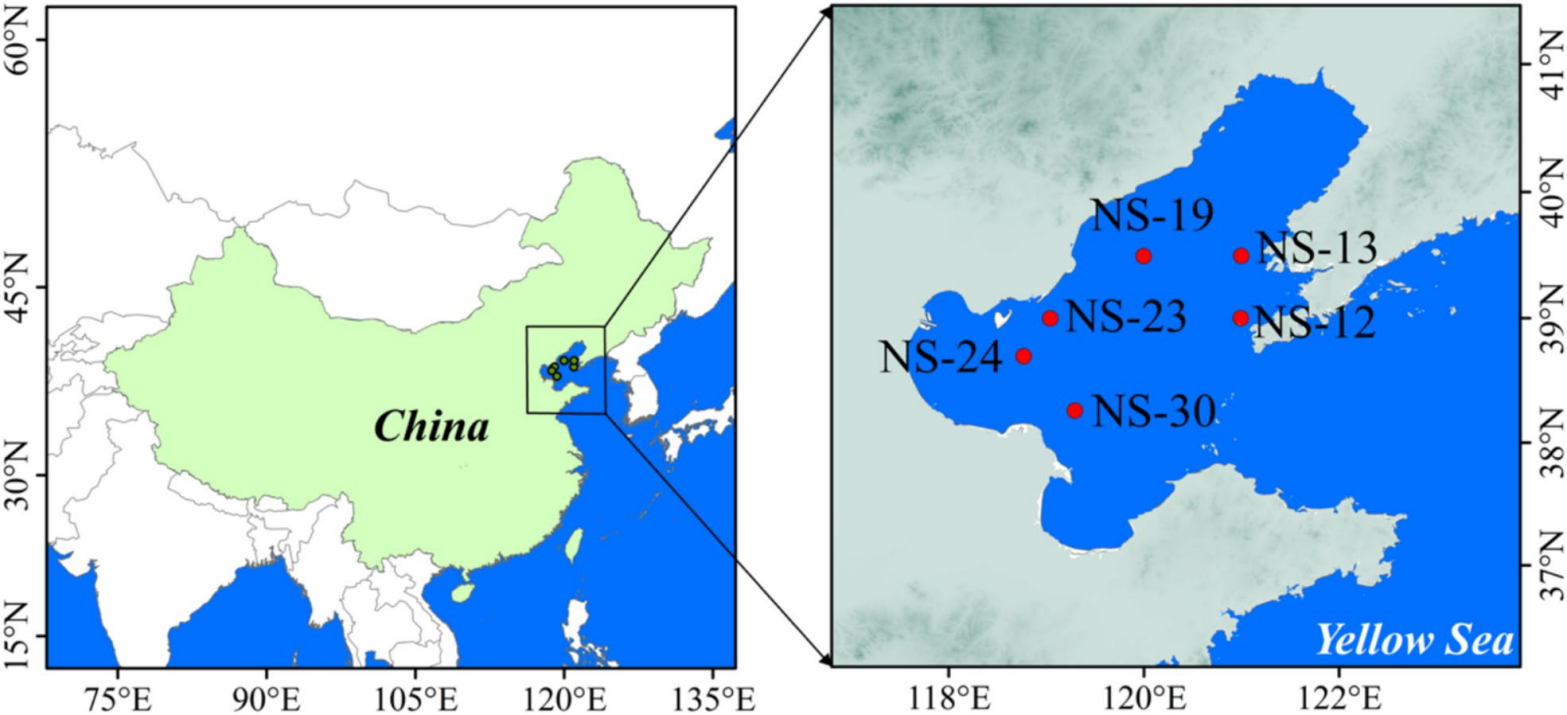
The sampling sites along the coastline of the Bohai Sea of China.

Bohai Sea (BS), China’s northernmost offshore sea, is a semi-enclosed sea area composed of the Liaodong Bay, the Bohai Bay, the Laizhou Bay and the Central Basin. Its special geographical location not only leads to the rapid development of various fisheries, aquaculture industries and the tourism industry, which in turn results in serious pollution, but also it receives numerous nourishing substances (e.g., ammonium) from the Yellow River, Liao River and other rivers. The unique natural and cultural factors have created a diverse environment. Moreover, they also provide an excellent opportunity to explore the characteristics of ammonia oxidizers within complicated biogeochemical settings. A total of six marine sediments were collected from the BS (NS-12, NS-13, NS-19, NS-23, NS-24, NS-30) (Figure 1). Sampling was conducted in May 2024. Surface sediment samples were collected using box-type mud buckets, transported to the laboratory in a cooler (0–4°C) for subsequent analyses, mixed thoroughly, and placed into zip-lock sterile plastics bags finally. And the composite samplings were divided into three parts. One subsample (1,080 g) was immediately determined for microbial activity (as detailed in 2.5). A small portion (72 g) was analyzed for determining sediment physicochemical characteristics (2.2), while the remaining (13.5 g) was stored at −20°C for molecular analysis (2.3 & 2.4).

### 2.2 Environmental factor analysis

Sediment pH was determined using a PHS-3C pH (Sartorius, China) meter with a sediment: water ratio of 1:2.5 (Zhang et al., 2023). Sediment KCl-extractable NH_4_^+^-N, NO_2_^−^-N and NO_3_^−^-N concentrations were extracted with a sediment: 2 M KCl ratio of 1: 7, and the supernatant was filtered through a 0.45 μm filter membrane. Ammonium, nitrite, and nitrate were determined with phenol-hypochlorite colorimetry, naphthalene amine colorimetry, and spectrophotometry, respectively, using a UV spectrophotometer (Liu et al., 2024). Total organic carbon (TOC) content was measured using a total organic carbon analyzer (TOC-L/SSM-5000A, Japan) using the sediment dried at 60°C. Total nitrogen (TN) content was analyzed by alkaline potassium persulfate digestion followed by UV spectrophotometry (Cheng et al., 2022). The microplastics (MPs) extracted from the sediment were analyzed using an LDIR imaging system (Agilent Technologies 8700 LDIR instrument, Germany), known for its high efficiency (Ourgaud et al., 2022).

### 2.3 DNA and RNA extraction

Total DNA was extracted from 0.25 g of sediment samples (wet weight) with the DNeasy^®^ PowerSoil^®^ Pro Kit (Mo Bio Laboratoties, Carlsbad, CA) according to the manufacturer’s protocols. Extraction and purification of total RNA from 2 g of sediment samples (wet weight) were carried out using the RNeasy^®^ PowerSoil^®^ Total RNA Isolation Kit (Mo Bio Laboratoties, Carlsbad, CA) following the manufacturer’s protocols, respectively.

### 2.4 Quantitative PCR at both genetic and transcriptional levels

cDNA was synthesized from the purified RNA using a QuantiTect^®^ Reverse Transcription Kit (Qiagen Germany). The obtained DNA and cDNA were used in subsequent steps. The obtained DNA and cDNA were carried out as templates for qPCR with the following primers: amoAF/amoAR for the AOA *amoA* gene (Francis et al., 2005), amoA1F/amoA2R for the AOB *amoA* genes (Rotthauwe et al., 1997) and Ntsp-amoA162F/Ntsp-amoA359R for the comammox *amoA* gene (Fowler et al., 2018) (Supplementary Table S2). The qPCR assays were performed in triplicate with the QuantiFluor^™^-ST Blue Fluorescence Quantification System (Promega). Each amplification was conducted in a 25-μL reaction system containing 12.5 μL of SYBR Green^®^ Premix Ex Taq^™^ II (TaKaRa, Japan), 10.5 μL of ddH_2_O, 0.5 μL of each primer, and 1 μL cDNA/DNA. The special thermal cycling steps were recorded in the Supplementary Table S2, respectively. After the amplification, a melting stage was added to obtain a melting curve. For each performance, positive control (standard plasmids), which was amplified by 10-fold serial dilution with primer pairs, and negative (sterile water) control were added to ensure that the qPCR assays were stable and uncontaminated. The abundance and transcript abundance of *amoA* genes were calculated following the standard curves generated with the standard plasmids containing archaeal or bacterial *amoA* genes.

### 2.5 Potential nitrification rates of AOA, AOB and comammox

The potential nitrification rates of AOA, AOB and comammox were measured through the double-inhibition method by using KClO_3_ (to suppress NOB activity) and 1-octyne (to suppress AOB activity) (Hynes and Knowles, 1983; Belser and Mays, 1980; Xu et al., 2011; Van Ginkel et al., 1995). First, fresh sediment (5 g) was added to each 60 mL serum bottle, which was sealed with rubber stoppers for the following three treatments. (1) Control group (treatment I), each serum bottle was added with 1.5 mL ultrapure water. (2) Nitrite oxidation-inhibition group (treatment II): 1.5 mL KClO_3_ (0.13 M) was added. (3) Sequential inhibition group (treatment III): 5 mL of KClO_3_ (0.13 M) and 2 kPa of 1-octyne were sequentially added to the serum bottles. The serum bottles were incubated in the dark, with samples taken at 0, 24, 48, 96 h in triplicate, respectively. NO_2_^−^-N and NO_3_^−^-N were extracted completely by 7 mL KCl (2 M) and shaken at 150 rpm for 30 min. The extracted solution was placed in 50 mL centrifugal tubes to determine NO_2_^−^-N and NO_3_^−^-N concentrations. The potential comammox rate was determined by comparing the nitrate accumulation (treatment I) and nitrite accumulation (treatment II), being represented as ∆NO_3_^−^-N (I) − ∆NO_3_^−^-N (II) − ∆NO_2_^−^-N (II) + ∆NO_2_^−^-N (I). To determine the rates of AOA and AOB, it was assumed that NO_2_^−^ was generated solely by AOA and AOB, and that no NO_2_^−^ was consumed by nitrite-oxidizing or denitrification processes in treatment II. The AOA rate was determined based on the NO_2_^−^ accumulation rate. By integrating this with the date from treatment II, the potential rate of AOB could subsequently be calculated (Wang et al., 2021).

### 2.6 Statistical and data analysis

Pearson’s correlation analysis and Mantel test were conducted to reveal the associations of AOA, AOB, and comammox between their *amoA* abundance, activity, and environmental factors. For the analysis mentioned above, the DNA abundance and cDNA abundance were logarithmic transformed. The statistical analysis and relative contributions to ammonia oxidation graph were analyzed with R (version 4.4.2). Other graphs were generated by the Prism 10 software.

## 3 Results and discussion

### 3.1 Physical and chemical properties of Bohai sediment samples

The physicochemical properties of the marine sediment samples in Bohai are shown in Supplementary Table S1. A total of six samples were collected from marine sediments in Bohai. Generally, all the sediments were slightly alkaline with pH larger than 7.67. The NH_4_^+^-N concentrations in the six marine sediments, sorted from the highest to the lowest, were NS-12 > NS-24 > NS-23 > NS-19 > NS-13 > NS-30, ranging from 0.82 to 2.52 mg/kg dry weight. The NO_2_^−^-N and NO_3_^−^-N contents ranged from 0.0059 to 0.1299 mg/kg dry weight and 5.20 to 32.97 mg/kg dry weight, respectively. TN had a similar trend to ammonia nitrogen, with the highest value (181.24 mg/kg dry weight) in NS-19 and the lowest (66.33 mg/kg dry weight) in NS-24. TOC varied from 5.42 to 10.35 mg/kg dry weight, with the lowest in NS-23 and the highest in NS-19. Furthermore, MPs were measured, ranging from 559.44 to 3340.33 particles/kg dry weight, with a substantial gap.

### 3.2 Total and active transcriptional abundance of the *amoA* gene in the marine sediments

**Figure 2:**
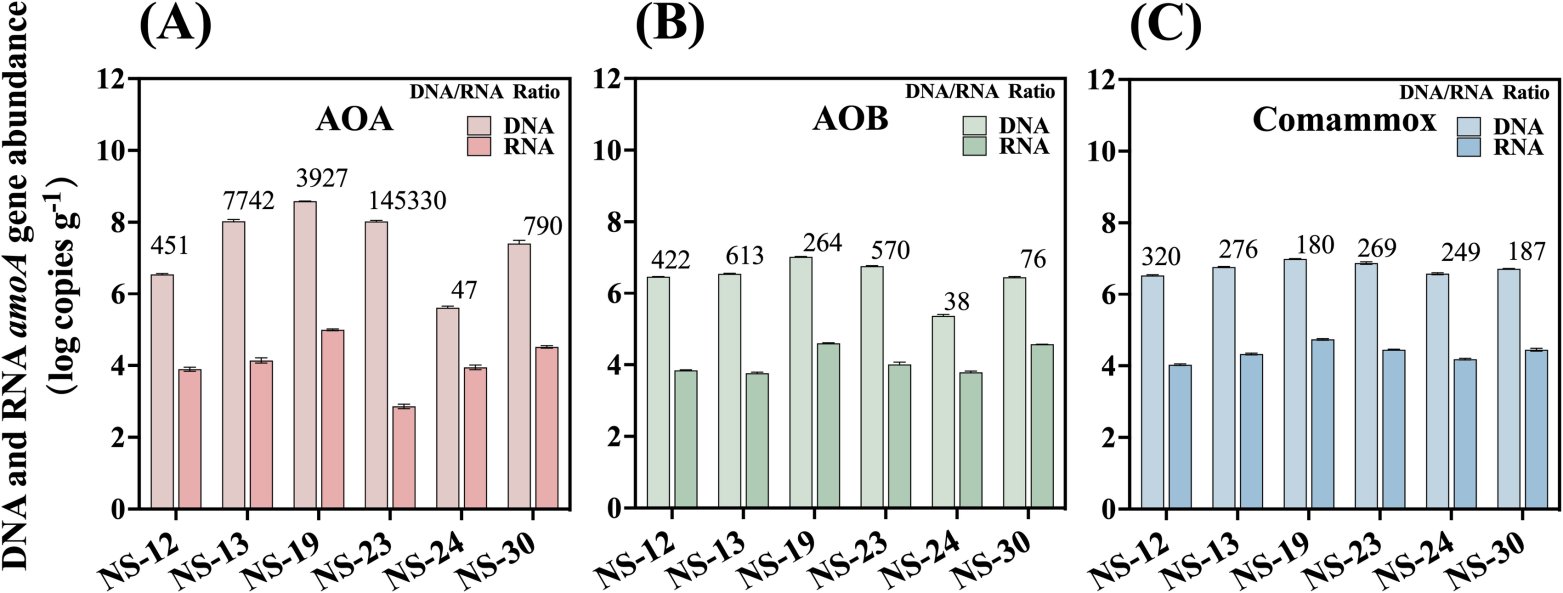
Abundance of *amo*A genes of AOA **(A)**, AOB **(B)** and comammox **(C)** at both DNA and RNA level in the marine sediment samples. The values displayed on the bars are DNA/RNA ratios. Error bars indicate standard deviation (*n* = 3).

The existence of ammonia oxidizers in marine sediment was studied by PCR amplification of the diagnostic gene *amoA*. The total abundance of AOA, AOB, and comammox ranged from 4.04 × 10^5^ to 3.85 × 10^8^, 2.30 × 10^5^ to 1.05 × 10^7^ and 3.43 × 10^6^ to 9.78 × 10^6^ copies g^−1^ dry sediment, respectively (Figure 2 and Supplementary Table S3). Among the sampling sites, one location stood out in terms of *amoA* gene abundance. Notably, NS-19 exhibited the highest total *amoA* gene abundance for all AOM (ammonia oxidizing microorganisms), while the location with the lowest abundance varied among AOA, AOB, and comammox.

Many researchers have studied the relationship between community structure and spatial distributions (Hu et al., 2015; Liu et al., 2023). However, it was reported that the abundance rather than community structure could be better account for the variation of nitrification rates (Hou et al., 2013). Moreover, it was evidenced that the variation of *amoA* gene abundances were explained more by spatial variation (Li et al., 2022). Therefore, spatial factors, such as geographic distance, should not be neglected when accounting for the large variation in AOA abundance. In our study, AOA possesses the highest *amoA* gene abundance in the marine sediments yet differing by four orders of magnitude with large spatial variation. Even though there was no significant difference (*p* > 0.05), the abundance of AOA in this region demonstrates remarkable stability in the face of environmental factors that we investigated, including salinity and ammonia. These factors, which are generally considered to have a significant impact on AOA, failed to show any significant influence in this study. This suggests that other factors, possibly unique to this region, may be playing a more dominant role in regulating AOA abundance.

The abundance for AOB in our finding was an order of magnitude less than that of river sediments (Ginawi et al., 2020), but the same as other marine sediments and mangrove ecosystems (Lipsewers et al., 2014; Liu et al., 2020). Based on the significant association between ammonium concentration and AOB *amoA* gene (Liu et al., 2018), high abundance of AOB was usually detected in environments with high ammonium concentration, such as in the marine sediments (Li et al., 2024) and black loam soil (Sun et al., 2021).

The distribution pattern of comammox resembled that of AOB. The abundance of comammox in our study was lower than that detected in the riparian sediments from Baiyangdian Lake (Wang et al., 2021). This might be attributed to the higher salinity in the marine compared to that in the riparian. Salinity typically played a crucial role in shaping archaeal and bacterial communities through influencing osmotic pressure, microbial respiration and microbial internal molecules, including microbial products, microbial enzyme and extracellular polymeric substance (Behera et al., 2017; Chen et al., 2017; Franklin et al., 2017; Wang et al., 2018). A higher salinity accompanied by high concentrations of Na^+^ and Ca^2+^ was reported to exert toxic effects on cells and affect the normal physiological and metabolic processes of microorganisms, thereby leading to a lower abundance of comammox (He et al., 2017; Santos et al., 2018). Previous studies have indicated that AOA tend to dominate in high-salinity environments (He et al., 2015). However, in our research, we found that salinity was not significantly correlated with the abundance of AOA, but it was associated with their activity further explored in 3.4.

To further explore the active transcription changes in ammonia oxidizers and their spatial distribution in the marine sediment, we quantified the active *amoA* gene at the RNA level, which will allow for a more accurate and clear definition of the ecological significance of AOM in nitrification processes. Previous studies on AOM in the marine sediment were mostly based on the DNA level and thus produced many contradictory conclusions (Fan et al., 2015; He et al., 2018). The transcriptional *amoA* genes abundance of AOA, AOB and comammox ranged from 7.31 × 10^2^ to 9.82 × 10^4^, 5.77 × 10^3^ to 3.98 × 10^4^ and 1.07 × 10^4^ to 5.44 × 10^4^ copies g^−1^ dry sediment, respectively, 2–6 orders of magnitudes lower than total functional gene abundances. The average abundance for AOA, AOB and comammox were 2.71 × 10^4^, 1.77 × 10^4^ and 2.63 × 10^4^ copies g^−1^ dry sediment, respectively, and their quantities differ slightly. The total abundance to transcription abundance ratio was relatively stable, ranging from 2 to 3 for AOB and comammox. Surprisingly, the ratio of AOA had a larger range of 2 to 5, which could have been attributed to the TOC concentration overall (*r* = 0.67, *p* < 0.05) and will be further examined.

### 3.3 Potential nitrification rates and relative contributions to ammonia oxidation of the three ammonia-oxidizers in marine sediments

**Figure 3:**
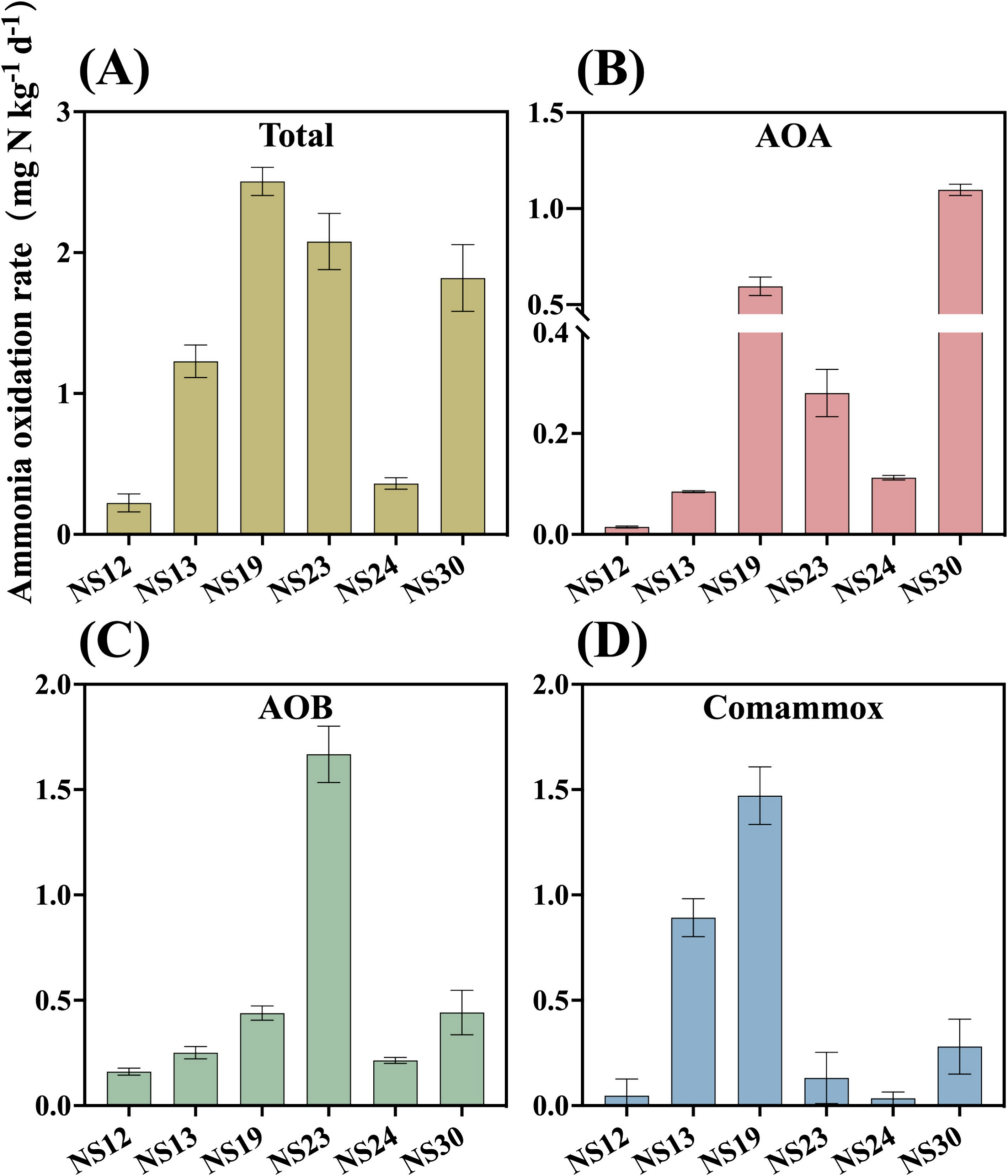
Rates of ammonia oxidation of AOA **(B)** AOB **(C)** comammox **(D)** and total of them **(A)** in the marine sediment samples. Error bars indicate standard deviation (*n* = 3).

**Figure 4:**
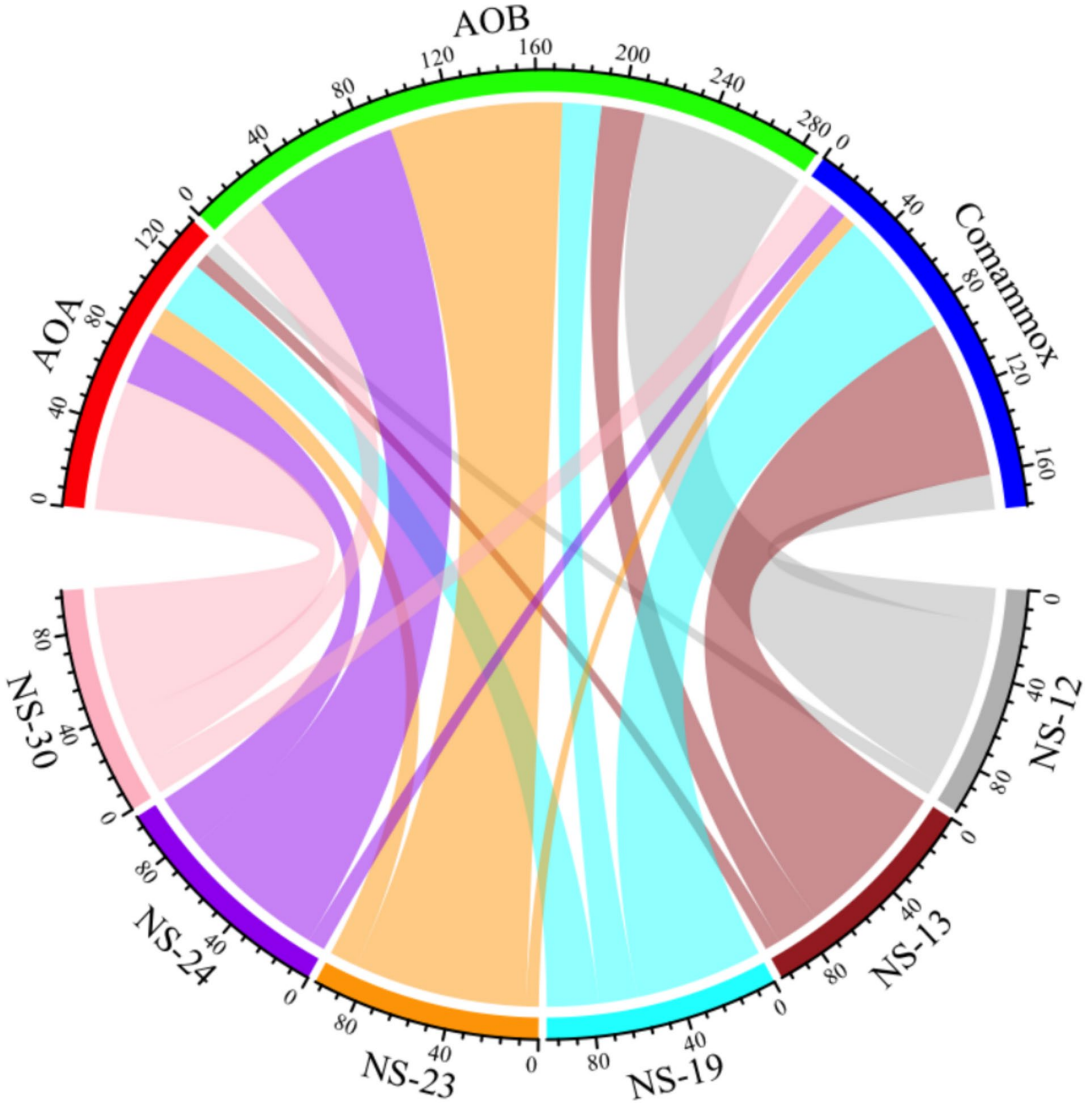
Relative contributions of AOA (red), AOB (green) and comammox (blue) to ammonia oxidation processes were drawn above.

To analyze the active ammonia oxidation process of ammonia oxidizers in the marine sediment, we determined the ammonia oxidation rate. The results showed that activity of AOA, AOB and comammox ranged from 0.0147 to 1.0976 mg N kg^−1^ dry sediment d^−1^, 0.1613 to 1.6675 mg N kg^−1^ dry sediment d^−1^ and 0.0337 to 1.4714 mg N kg^−1^ dry sediment d^−1^, respectively. The highest rate of AOB was observed at NS-23, with AOB making major contributions in NS-12 (77%), NS-23 (80%), NS-24 (68%). However, comammox and AOA had significant contributions at NS-13 (73%), NS-19 (59%) and NS-30 (60%) (Figures 3, 4 and Supplementary Table S4), which contrasted with previous findings that AOA was dominant (Li et al., 2022; Zhao et al., 2024). It was reported that the abundance of AOB was higher than that of AOA in the marine surface sediments, leading to the hypothesis that AOB might play a primary role in ammonia oxidation process (He et al., 2015). However, Zheng et al. (2014) found that despite the higher abundance of AOB compared to of AOA in the intertidal sediments of the Yangtze Estuary, no difference between the activity in microcosms with without AOB, indicating the AOA contributed more to the nitrification potential. In the present study, AOA possessed the higher total abundance but lower contribution to ammonia oxidation, while AOB was the opposite. Researchers have attempted to reveal the relative contributions of AOA or AOB to nitrification through the relationships between *amoA* gene abundance and activity as well as found that AOB transcription abundance positively correlated with potential nitrification rates in the coastal microbial ecosystems (Fan et al., 2015), but the relationships are intricate and uncertain (Bernhard et al., 2010; Wang et al., 2019). It is incomprehensive and limited to calculate the relative role of AOM based on the total *amoA* gene abundances or the activity. Therefore, the relative importance of AOA, AOB and comammox in nitrification should be focus on the relative contributions to ammonia oxidation and transcription abundance (Li et al., 2022).

Generally, one of the most important factors determining the activity of AOA and AOB in soil is pH, with AOA being more tolerant to acidic or nutrient-poor environments (Nicol et al., 2008; Erguder et al., 2009). Our study found a significant positive correlation between comammox and pH as well (*r* = 0.55, *p* < 0.05), consistent with previous study indicating that comammox preferred to grow in slightly alkaline soils (Xu et al., 2020).

Pearson correlation analysis showed that NO_3_^−^ concentration significantly affected the relative contribution of AOA (*r* = 0.52, *p* < 0.01). Prior to this study, no study has reported that nitrate can affect the relative contribution of AOA among ammonia-oxidizing microorganisms. Most studies have revealed significant correlations between nitrate concentration and AOA activity (Ye et al., 2024) or abundance (Wu et al., 2023). Our results indicated that nitrate not only affected the absolute abundance and activity of AOA, but also increased the relative contribution of AOA among ammonia-oxidizing microorganisms as nitrate raised.

### 3.4 Associations among total abundance, active transcription abundance, activity and environmental factors

**Figure 5:**
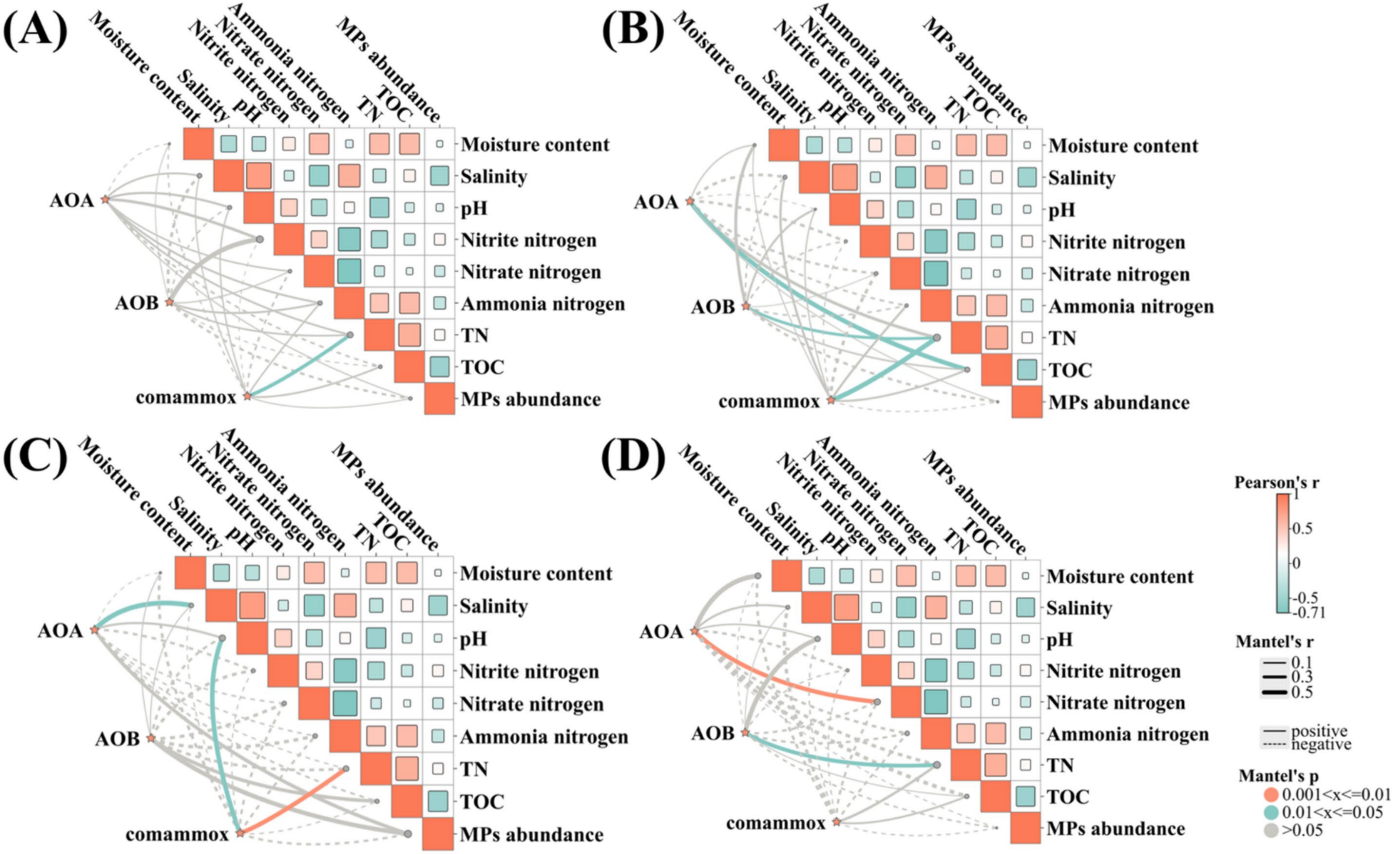
Correlation analysis and Mantel test show the correlations among DNA **(A)**, RNA **(B)**, rate of ammonia oxidation **(C)**, relative contribution **(D)** and environmental factors in the marine sediment samples.

Due to the particularity of the marine system, the process of marine nitrogen cycling exhibits obvious spatial heterogeneity. This study investigated the physicochemical factors affecting AOA, AOB and comammox. Pearson’s correlation showed a strong correlation among the abundance, activity, relative contribution and environmental factors. Scholars often indicate that AOA played a dominant role in high-salt environments, which partly explains the relatively higher abundance of AOA in the marine environment (Li et al., 2022). While our result did not indicate a significant relationship between salinity and the abundance of AOA, a positive relationship was observed between salinity and AOA activity (*r* = 0.59, *p* < 0.05) (Figure 5 and Supplementary Table S5).

TN displayed a significant positive correlation with the total abundance of comammox (*r* = 0.62, *p* < 0.05), the transcription abundance of AOB (*r* = 0.38, *p* < 0.05) and comammox (*r* = 0.72, *p* < 0.05), the activity of comammox (*r* = 0.53, *p* < 0.01), and the relative contribution of AOB (*r* = 0.47, *p* < 0.05). Interestingly, TN was solely correlated with comammox and AOB, with no relation to AOA. This aligns with previous studies showing that a higher total abundance of AOM was observed in higher TN concentration (Zhang et al., 2022; Feng et al., 2024). It was speculated that more TN would partly increase the substrate, providing more nitrogen sources and participating in the energy metabolism of AOM.

In this study, taking comammox as an example, it was found that, when the abundances on DNA and RNA levels were quite similar from the six samples of marine sediment, there were significant differences in activity. We may infer that TN and pH may play a crucial role in the process of RNA translation, as well as in the synthesis of proteins and the regulation of their biological activities. It remained to be studied how environmental factors affect the ecological niche distribution of ammonia-oxidizing microorganisms from the perspective of molecular biology. Nevertheless, this study provides a preliminary understanding that environmental factors such as total nitrogen and pH play a certain positive role in the abundance and activity of comammox and AOB.

## 4 Conclusion

This study investigated the abundance at DNA and RNA levels, the ammonia oxidation rates of ammonia oxidizers, and their correlations with environmental factors in the Bohai sediments to qualify the spatial variations. At the genetic level, AOA was the most abundant ammonia oxidizer. TOC significantly affected the transcript abundance, while multiple factors had significant influence on ammonia oxidation rates. TN had an impact on total abundances, active transcript abundances and activity. Correlation analysis showed that no single parameter was likely to determine the whole abundance and activity. Therefore, this study provided comprehensive insights into the microbial mechanisms driving nitrification in marine environments.

## Data Availability

The original contributions presented in the study are included in the article/Supplementary material, further inquiries can be directed to the corresponding authors.
