# Supplementary material for: Genetic and transcriptional profiles of ammonia oxidizing communities in Bohai sediments: abundance, activity, and environmental correlations
Source: Front Microbiol. 2025 May 27;16:1611213. doi: 10.3389/fmicb.2025.1611213 (PMC12150803; doi:10.3389/fmicb.2025.1611213)
Supplement: Supplementary file 1 [file Data_Sheet_1.pdf]

# *Supplementary Material*

**Table S1.** Physico-chemical characteristics of the sediment collected from the Bohai Sea.

| Sampling sites | Longitude | Latitude | Moisture content (%) | Salinity (psu) | pH | $NO_2^-$ (mg/Kg) | $NO_3^-$ (mg/Kg) | $NH_4^+$ (mg/Kg) | TN (mg/Kg) | TOC (g/Kg) | MPs abundance (Particles/Kg d.w.) |
|---|---|---|---|---|---|---|---|---|---|---|---|
| NS-12 | 120.99° | 39.00° | 30.19 | 30.60 | 7.85 | 0.0079 | 5.20 | 2.29 | 66.38 | 8.67 | 559.44 |
| NS-13 | 121.00° | 39.50° | 29.96 | 29.17 | 7.73 | 0.0234 | 5.59 | 1.39 | 106.74 | 7.44 | 2308.01 |
| NS-19 | 120.00° | 39.49° | 50.57 | 29.54 | 7.75 | 0.0059 | 6.15 | 2.52 | 181.24 | 10.35 | 2304.73 |
| NS-23 | 119.04° | 39.00° | 30.44 | 29.48 | 7.79 | 0.0087 | 5.40 | 1.66 | 85.77 | 5.42 | 3340.33 |
| NS-24 | 118.77° | 38.70° | 44.77 | 29.40 | 7.84 | 0.1299 | 22.85 | 0.82 | 66.33 | 7.28 | 2442.98 |
| NS-30 | 119.29° | 38.26° | 46.19 | 28.87 | 7.67 | 0.0138 | 32.97 | 1.09 | 106.26 | 8.06 | 1440.10 |

**Table S2.** Primers used in this study.

| Target gene | Primer name | Primer sequences (5'-3') | Length (bp) | Thermal profiles | Reference |
|---|---|---|---|---|---|
| AOA *amoA* | amoAF/amoAR | ATGGTCTGGYTWAGACG<br>GCCATCCABCKRTANGTCCA | 629 | 5 min at 95 °C, 35 cycles consisting of 30 s at 95 °C, 1 min at 50 °C and 1 min at 72 °C ,5 min at 72 °C | Francis et al., 2005 |
| AOB *amoA* | amoA1F/amoA2R | GGGGTTTCTACTGGTGGT<br>CCCCTCKGSAAAGCCTTCTTC | 491 | 3 min at 95 °C, 35 cycles consisting of 30 s at 95 °C, 30 s at 55 °C and 30 s at 72 °C ,5 min at 72 °C | Rotthauwe et al., 1997 |
| comammox *amoA* | Ntsp-amoA162F<br>Ntsp-amoA359R | GGATTTCTGGNTSGATTGGA<br>WAGTTNGACCACCASTACCA | 198 | 5 min at 94 °C, 40 cycles consisting of 30 s at 94 °C, 30 s at 48 °C and 1 min at 72 °C | Fowler et al., 2018 |

**Table S3.** Quantitative analysis of AOA, AOB and comammox *amoA* gene in the 6 sediment samples from the Bohai Sea.

| Sample | DNA and RNA *amoA* gene abundance (copies $g^{-1}$) | | | | | |
|---|---|---|---|---|---|---|
| | AOA | | AOB | | comammox | |
| DNA/RNA | DNA | RNA | DNA | RNA | DNA | RNA |
| NS-12 | $3.51 \times 10^6$ | $7.92 \times 10^3$ | $2.92 \times 10^6$ | $6.93 \times 10^3$ | $3.43 \times 10^6$ | $1.07 \times 10^4$ |
| NS-13 | $1.07 \times 10^8$ | $1.39 \times 10^4$ | $3.51 \times 10^6$ | $5.77 \times 10^3$ | $5.87 \times 10^6$ | $2.13 \times 10^4$ |
| NS-19 | $3.85 \times 10^8$ | $9.82 \times 10^4$ | $1.05 \times 10^7$ | $3.98 \times 10^4$ | $9.78 \times 10^6$ | $5.44 \times 10^4$ |
| NS-23 | $1.05 \times 10^8$ | $7.31 \times 10^2$ | $5.80 \times 10^6$ | $1.03 \times 10^4$ | $7.56 \times 10^6$ | $2.81 \times 10^4$ |
| NS-24 | $4.04 \times 10^5$ | $8.92 \times 10^3$ | $2.30 \times 10^5$ | $6.13 \times 10^3$ | $3.82 \times 10^6$ | $1.53 \times 10^4$ |
| NS-30 | $2.60 \times 10^7$ | $3.30 \times 10^4$ | $2.85 \times 10^6$ | $3.73 \times 10^4$ | $5.23 \times 10^6$ | $2.80 \times 10^4$ |

**Table S4.** Spatiotemporal variations of rate of AOA, AOB and comammox in the 6 sediment samples from the Bohai Sea.

| | Ammonia oxidation rate ($mg\ N\ kg^{-1}\ d^{-1}$) | | | | Relative of contribution (%) | | |
|---|---|---|---|---|---|---|---|
| | AOA | AOB | comammox | sum | AOA | AOB | comammox |
| NS-12 | 0.0147 | 0.1613 | 0.0337 | 0.2097 | 7.00 | 76.94 | 16.06 |
| NS-13 | 0.0848 | 0.2513 | 0.8923 | 1.2284 | 6.90 | 20.46 | 72.64 |
| NS-19 | 0.5955 | 0.4391 | 1.4714 | 2.5060 | 23.77 | 17.52 | 58.71 |
| NS-23 | 0.2801 | 1.6675 | 0.1311 | 2.0787 | 13.48 | 80.22 | 6.31 |
| NS-24 | 0.1123 | 0.3088 | 0.0344 | 0.4555 | 24.65 | 67.80 | 7.55 |
| NS-30 | 1.0976 | 0.4419 | 0.2801 | 1.8196 | 60.32 | 24.29 | 15.40 |

**Table S5.** Pearson's correlation coefficients between AOA, AOB and comammox abundance, activity, relative contribution and physico-chemical characteristics.

| | | | Moisture content | Salinity | pH | Nitrite nitrogen | Nitrate nitrogen | Ammonia nitrogen | TN | TOC | MPs abundance |
|---|---|---|---|---|---|---|---|---|---|---|---|
| Abundance | AOA | DNA | 0.4500 | 0.3111 | 0.1556 | 0.1472 | 0.2764 | 0.1861 | 0.3028 | 0.7278 | 0.4139 |
| | | RNA | 0.3264 | 0.7569 | 0.5167 | 0.8958 | 0.7833 | 0.7653 | 0.2000 | **0.0431**$^{*}$ | 0.4569 |
| | AOB | DNA | 0.3931 | 0.9264 | 0.6736 | 0.0764 | 0.2972 | 0.1722 | 0.2347 | 0.4500 | 0.7306 |
| | | RNA | 0.1347 | 0.6347 | 0.1653 | 0.7264 | 0.3028 | 0.7569 | **0.0486**$^{*}$ | 0.2792 | 0.9569 |
| | comammox | DNA | 0.6625 | 0.3708 | 0.2806 | 0.4486 | 0.8278 | 0.2125 | **0.0139**$^{*}$ | 0.2125 | 0.4167 |
| | | RNA | 0.3694 | 0.3056 | 0.3778 | 0.4014 | 0.9722 | 0.1653 | **0.0167**$^{*}$ | 0.2306 | 0.5764 |
| Activity | AOA | | 0.3597 | **0.0403**$^{*}$ | 0.2167 | 0.7236 | 0.5801 | 0.6403 | 0.4778 | 0.6625 | 0.1069 |
| | AOB | | 0.3472 | 0.3972 | 0.7500 | 0.9528 | 0.7667 | 0.9181 | 0.7611 | 0.2514 | 0.1139 |
| | comammox | | 0.6472 | 0.6153 | **0.0375**$^{*}$ | 0.6611 | 0.9917 | 0.7556 | **0.0028**$^{**}$ | 0.6306 | 0.9222 |

| | | | | | | | | | | |
|---|---|---|---|---|---|---|---|---|---|---|
| Relative contribution | AOA | 0.0542 | 0.2292 | 0.3292 | 0.9389 | **0.0056**$^{**}$ | 0.7958 | 1.0000 | 0.9944 | 0.5292 |
| | AOB | 0.1764 | 0.4542 | 0.0958 | 0.9569 | 0.7681 | 0.6625 | **0.0139**$^{*}$ | 0.2958 | 0.6778 |
| | comammox | 0.6819 | 0.9653 | 0.6431 | 0.6042 | 0.9639 | 0.7722 | 0.1625 | 0.2556 | 0.5528 |

Significance： $^{**}$ $P < 0.01$, $^{*}$ $P < 0.05$

*. Correlation is significant at the 0.05 level.

**. Correlation is significant at the 0.01 level.
